# Dietary Intakes and Nutritional Issues in Neurologically Impaired Children

**DOI:** 10.3390/nu7115469

**Published:** 2015-11-13

**Authors:** Francesca Penagini, Chiara Mameli, Valentina Fabiano, Domenica Brunetti, Dario Dilillo, Gian Vincenzo Zuccotti

**Affiliations:** Pediatric Department, V. Buzzi Children’s Hospital, University of Milan, via Castelvetro 32, 20154 Milan, Italy; chiara.mameli@unimi.it (C.M.); valentina.fabiano@unimi.it (V.F.); domenica.brunetti@unimi.it (D.B.); dario.dilillo@icp.mi.it (D.D.); gianvincenzo.zuccotti@unimi.it (G.V.Z.)

**Keywords:** neurological impairment, children, dietary intake

## Abstract

Neurologically impaired (NI) children are at increased risk of malnutrition due to several nutritional and non-nutritional factors. Among the nutritional factors, insufficient dietary intake as a consequence of feeding difficulties is one of the main issues. Feeding problems are frequently secondary to oropharyngeal dysphagia, which usually correlates with the severity of motor impairment and presents in around 90% of preschool children with cerebral palsy (CP) during the first year of life. Other nutritional factors are represented by excessive nutrient losses, often subsequent to gastroesophageal reflux and altered energy metabolism. Among the non-nutritional factors, the type and severity of neurological impairment, ambulatory status, the degree of cognitive impairment, and use of entiepileptic medication altogether concur to determination of nutritional status. With the present review, the current literature is discussed and a practical approach for nutritional assessment in NI children is proposed. Early identification and intervention of nutritional issues of NI children with a multidisciplinary approach is crucial to improve the overall health and quality of life of these complex children.

## 1. Introduction

Nutritional status has a significant impact on overall health and quality of life in children with neurodevelopmental disabilities; both under and over nutrition generally lead to increased health care use and reduced participation in educational and social activities. Malnutrition is frequently associated with impairment of linear growth, reduced peripheral circulation and wound healing, increased spasticity and irritability. The overall prevalence of malnutrition in NI children is difficult to estimate, due to the heterogeneity of neurological disorders. The majority of scientific literature on nutrition in NI children has focused on the population with cerebral palsy (CP) in which malnutrition has been observed in 46%–90% of cases [1]. Etiology of malnutrition in NI children is multifactorial including both nutritional and non-nutritional factors. Among the nutritional factors, the main is represented by inadequate dietary intake as a consequence of gastrointestinal disorders including oral motor dysfunction, gastroesophageal reflux and constipation. Among non-nutritional factors, the type and severity of underlying neurological disability, influencing ambulatory and cognitive status, and antiepileptic use are crucial factors involved in determination of nutritional status.

## 2. Nutritional Factors

### 2.1. Dietary Intake

Inadequate dietary intake is one of the major contributors to poor nutritional status and altered growth in NI children. For the present review, a search of the literature using PubMed from 1991 to 2015 was performed with the following search terms “dietary intakes”, “nutrient intakes”, “feeding difficulties”, “neurologically impaired children” and “cerebral palsy”. One hundred articles were reviewed. Of these, we selected 15 articles that included: (1) pediatric data (age < 18 years); (2) data on oral dietary intake and feeding problems, obtained from clinical studies with information collected either retrospectively from medical records or prospectively in cross-sectional studies with food diaries, feeding histories and parental interviews. Several studies have shown reduced energy and nutrient intakes in children with motor disability [2,3,4,5,6,7,8,9,10,11,12]. In a study conducted by Kilpinen-Loisa *et al.* [7], 54 children with motor disability (CP in 59% of cases, median age 10.9 years) were assessed for nutrient intakes by means of a three-day food diary. The median energy intake was 76% of the recommended dietary intake and <80% in more than half of children (57%). Of the total energy, 17% was from protein, 32% from lipids and 50% from carbohydrates. The median intake of vitamin D was low, around 76% of recommended age-specific dietary intake. Children with total energy intake <80% of recommendations were more severely disabled and presented with significantly lower median height z-scores compared to children with total energy intake ≥80% of the age-specific recommendations (Gross Motor Function Classification Scale (GMFCS) scores 3.8 *vs*. 3.0, *p* = 0.011 and height z-scores −1.5 *vs.* −0.8, *p* = 0.02).

In a study conducted by Lopes *et al.* [11] on dietary patterns of 90 children with CP aged 2–13 years, dietary assessment was performed using the 24-h recall method and a Food Frequency Questionnaire. Children using nasogastric or gastrostomy feeding tube were excluded. In the interview, parents were also asked about difficulty in chewing and/or swallowing solid foods and the number of meals consumed per day. Difficulty in chewing solid food was observed in 23 (26%) cases, and for swallowing, in eight (9%) cases. Children with diplegia had no difficulty in chewing and swallowing, while the quadriplegic children had a higher prevalence of chewing (41%) and swallowing (12.8%) problems. The percentage of total energy coming from macronutrients showed that there was a dietary pattern low in carbohydrates (52%), adequate in protein (53%), and high in lipids (43%). The analysis according to the type of CP showed, in the group with hemiplegia, high intake of lipids (58%) and inadequate intake of carbohydrate and protein (50%). In the group with tetraplegia, 64% showed high lipid intake, 62%, low carbohydrate intake and 46% low protein intake. The average energy consumption showed no significant difference in children with the various types of CP. The analysis according to the age showed that in the age range 2–3 years, the average intake was in agreement with the recommendations; in the age range 4–6 years, the groups with hemiplegia and tetraplegia presented means below the lower limits of recommendation. In the age range 9–13 years, the group with tetraplegia showed average energy intake below the recommended. The study concluded for a high prevalence of inadequate dietary patterns in children with CP [11].

In a more recent study, Sangermano *et al.* [12] studied daily calorie intake, by means of a three-day food diary, in 30 children with neuromotor disabilities aged 2–15 years. The study showed that the average daily calorie intake was particularly low in 43% of patients, according to the energy requirements calculated with the specific formula for children with motor disability. The macronutrient intakes of these children were unbalanced, showing an increased intake of lipids and protein (lipids 37% and protein 17% of total energy intake) and reduced intake of carbohydrates (46% of total energy intake). Table 1 summarizes the main findings of the studies focusing on feeding problems and dietary intakes in NI children.

**Table 1:** Main studies on dietary intakes in neurologically impaired children.

Author-Year-Country	Population	Method	Main Findings
Thommessen M. *et al.* 1991, Norway [2]	Disabled children aged 1–16 years (*n* = 221)	Energy and nutrient intakes assessed with a 4-day food record.	Children with feeding problems or alternative feeding practices had lower energy and nutrient intakes than did children without these factors.
Hals J. *et al.* 1996, Norway [3]	Severely neurologically impaired children age range 2–13 years (*n* = 13)	Energy and nutrient intakes assessed with a 4-day food record.	Low dietary intakes of energy and of several nutrients with corresponding low hemoglobin values and serum values of ferritin, selenium and vitamins E and D in the study population.
Dahl M. *et al.* 1996, Norway [1]	Children with CP (*n* = 35, median age 8 years).	Parental interviews and medical records to assess feeding problems.	60% of children (21/35) reported to have daily feeding problems.
Reilly S. *et al.* 1996, United Kingdom [13]	Children with CP (*n* = 49, median age range 12–72 months).	Parental interview and video recording of mealtime to assess feeding problems.	Sucking (57%) and swallowing (38%) problems common in the first 12 months of life. 80% of children fed non-orally at least on one occasion.
Hals J. *et al.* 2000, Norway [4]	Severely neurologically disabled children age range 2–13 years (*n* = 13)	Dietary intakes assessed with a 4-day food record.	Low intake of essential fatty acids (EFA) and low serum concentrations of several EFA compared to those of a reference group of children.
Sullivan P.B. *et al.* 2000, United Kingdom [5]	Children with cerebral palsy age range 4.2–13.1 years (*n* = 271)	Questionnaire regarding gastrointestinal and feeding problems	Feeding problems were prevalent: 89% needed help with feeding and 56% choked with food; 20% described feeding as stressful and unenjoyable. Prolonged feeding time was reported by 28%.
Gangil A. *et al.* 2001 [6]	Children with CP (*n* = 100, age range 1–9 years)	Feeding problems assessed by observing feeding session.	Oral motor dysfunction observed in all cases. Spastic quadriplegic CP and hypotonic patients had significantly lower feeding skill scores. Inability to self feed in 16% of cases, swallowing problems in 19% of cases, drooling in 20% of cases.
Kilpinen-Loisa P. *et al.* 2009, Finland [7]	Children with motor disabilities (*n* = 54, age range 5–15.5 years).	Parental interview to assess feeding problems. A 3-day food diary to assess nutrient intake.	20% (11/54) reported to have feeding problems. Low intake of energy in 57% of cases, low intake of vitamin D on average 76% of recommendations, low intake of iron on average 87% of recommendations, poor intake of fiber on average only half of recommendations.
Hillesund E. *et al.* 2007, Norway [8]	Children with CP (*n* = 36, aged 1.5–17 years)	A 4-day food diary to assess micronutrient intake	Low intake of iron, folates, niacin, calcium, vitamin E and vitamin D was common even in those receiving nutritional supplements.
Grammatikopoulou M.G. *et al.* 2009, Greece [9]	Children with CP (*n* = 16, median age 10.1 ± 2.9 years) and their healthy siblings.	A 3-day food diary to assess nutrient intake.	Low energy intake in children with CP, covering 74.6% of their energy requirements. No differences in macronutrient distribution between CP children and healthy siblings. Subjects with CP had low vitamin A, biotin, folate, vitamin K, and copper intakes.
Calis E.A.C. *et al.* 2010, Netherlands [10]	Children with generalized cerebral palsy and intellectual disability (*n* = 176, median age 10 years).	A 7-day food diary to assess nutrient intakes.	Low intake of energy (62% of recommendations), calcium (87% of recommendations), vitamin A (77% of recommendations), vitamin D (73% of recommendations), vitamin B6 (13% of recommendations) and folates (78% of recommendations).
Walker J.L. *et al.* 2012, Australia [14]	Children with CP (*n* = 73, aged 2.8 ± 0.9 years), 16 typically developing children (TDC).	A validated 3-day weighed food record to assess nutrient intakes.	No significant differences in energy intakes between CP and TDC children. In CP group, considering for gross motor functional ability non-ambulant children had lower energy intakes compared to ambulant children (*p* < 0.01). Macronutrient composition of the diet was similar for all children. Protein intake was above the national recommended intake for all children.
Lopes P.A.C. *et al.* 2013, Brasil [11]	Children with CP (*n* = 90, age range 2–12.8 years).	Parental interview for feeding difficulties. A 24-h recall and food frequency questionnaire.	Prevalence of chewing and swallowing problems in children with spastic quadriplegia 41% and 12.8% respectively. Dietary pattern with low in carbohydrates (52%), adequate in protein (53%), and high in lipids (43%). Average energy intake below recommendations in children aged 9–13 years.
Sangermano M. *et al.* 2014, Italy [12]	Children with psychomotor developmental delay (*n* = 30, aged 2–15 years).	A 3-day food diary to quantify calorie and nutrient intake.	Low daily calorie intake in 43.3% of cases with unbalanced macronutrients: low in carbohydrates and high in lipids and protein (according to the Italian recommended daily intake of energy and nutrients).
Benfer K.A. *et al.* 2015, Australia [15]	Children with CP (*n* = 99, aged 18–36 months).	A 3-day weighed food record to assess dietary intake.	Energy intake of children decreased with poorer gross motor function. Food/fluid texture modifications occurred in 39% of cases and this proportion increased as gross motor function declined. Children on average had 50% of intake as fluid, which was most commonly unsafely swallowed.

### 2.2. Micronutrient Deficiencies

The above-described inadequate dietary intakes, besides from putting children at risk of calorie–protein malnutrition and reduced linear growth, may also lead to micronutrient deficiencies. Micronutrients are important for many metabolic pathways; specific or generalized micronutrient deficiencies may cause symptoms, which are difficult to distinguish from the general neurologic impairment of NI children. Low micronutrient levels may indeed affect cognition, behavior, social interaction, developmental outcomes and hence quality of life. A Norwegian study conducted by Hillesund *et al.* investigated micronutrient status in 36 children with CP aged 1.5–17 years [8]. Micronutrient intake was assessed with a four-day food diary and micronutrient status with laboratory analysis of micronutrient concentrations was performed. A low micronutrient intake was common in both children receiving (*n* = 16) and not receiving nutritional supplements (*n* = 20). Approximately 50% of all children had a low iron intake. Among the non-supplemented children, 80% (16/20) had low vitamin D intake, 65% (13/20) low niacin intake, 60% (12/20) low iron intake, 45% (9/20) low vitamin E intake, 40% (8/12) low folate intake, and 35% (7/20) low calcium intake. The 16 supplemented children had higher mean intakes of most vitamins and minerals, but insufficient intakes of folates, iron, magnesium and vitamin D was still observed in some patients. With regards to laboratory analysis, the children who received multi-vitamin supplements presented with higher concentrations of thiamine and cobalamin compared to those who did not receive supplements. Of all children, 22.2% (8/36) had low serum folates, 16.6% (6/36) had low serum vitamin E, 13.8% (5/36) had depleted iron stores (ferritin < 12 μg/L), 8.3% (3/36) had low cobalamin levels and 5.5% (2/36) had low zinc concentrations. Overall, the study found low dietary intakes and biochemical deficiency of several micronutrients in children with CP, especially in those not receiving nutritional supplements. More recently, a study conducted by Kalra *et al.* compared micronutrient levels in 50 children with CP (aged 2–12 years) and neurologically normal controls matched for age and sex [16]. The serum levels of iron (12.6 ± 5.9 and 20.9 ± 3.3 μmoL in CP and in controls respectively, *p* < 0.01), copper (106.2 ± 38.3 µg/dL in CP and 128.8 ± 20.2 in controls, *p* < 0.001) and magnesium (1.97 ± 0.4 in CP and 2.2 ± 0.3 mg/dL in controls) were significantly lower in children with CP compared to the control group. Levels of zinc were lower though difference was not statistically significant. The present study confirms that biochemical deficiency of micronutrients is common in children with CP, indicating that dietary intakes of vitamins and minerals are often too low to balance needs in this population. Furthermore, also children who are exclusively tube fed, may develop nutrient deficiencies, because enteral formulas provide adequate amounts of micronutrients only when volumes consumed meet their age-related daily recommended intakes for energy. Many NI children require lower energy intakes posing them at risk for low micronutrient intake [17]. Iron deficiency anemia is a frequent complication in NI children due to low iron intake. Papadopoulos *et al.* found a high incidence of anemia in patients with CP (*n* = 108, age range 8–29 years); in fact, anemia was found in 87% and iron deficiency was found in 95.6% of subjects on a liquid diet [18]. Selenium deficiency is another issue that can be encountered in NI children who receive long-term enteral nutrition, as some types of medical nutrition products do not contain adequate doses of selenium. Selenium is an essential trace element and a component of selenoproteins. A study conducted by Etani *et al.* analyzed serum selenium levels of children and adolescents with neurological disabilities (*n* = 71, age range 7 months–20 years) and/or intestinal dysfunction (*n* = 24, age range 7 months–20 years) who received either parenteral nutrition and/or enteral nutrition for more than three months. Twenty-eight (29%) patients showed serum selenium levels below 4 μg/dL. Five patients whose serum selenium levels were below 2 μg/dL presented clinical manifestations including hair browning (*n* = 5), macrocythemia (*n* = 4), nail whitening (*n* = 3) and cardiac dysfunction (*n* = 1) [19]. Carnitine deficiency is relatively common in children with epilepsy. Carnitine is a water-soluble quaternary amine with important intracellular functions, but is only biologically active in the l-isoform. Approximately 75% of carnitine is obtained from the diet and the remainder from endogenous biosynthesis; carnitine deficiency can cause complications such as muscle weakness, cardiopathy and in severe cases also hypoglycemia, abdominal pain, vomiting and enlarged liver. The risk factors for carnitine deficiency are reported to include multiple antiepileptic drug therapy (including valproic acid), young age (<10 years), neurological disability (intellectual disability, cerebral palsy and microcephaly), a diet deficient in meat and dairy products, tube feeding or parenteral nutrition [20,21,22]. Recently, Fukuda *et al.* [23] have conducted a study in children with epilepsy (*n* = 65) and compared plasma carnitine concentrations with 26 age-matched controls. Carnitine deficiency was found in approximately 17% of patients with epilepsy and was significantly associated with use of carnitine-free enteral formulas only by tube feeding, number of antieplipetic drug medications, body weight, body height and gross motor function [23].

### 2.3. Altered Energy Requirements

Alterations in energy requirements (ERs) frequently occur in NI children and are an important determinant of nutritional status. ERs for children with severe neurodisabilities such as CP are different from those recommended for neurologically normal children due to the influence of many factors altering their resting energy expenditure (REE). First, because ambulatory status and characteristics of motor impairment (type, distribution and severity) influence movement patterns (choreoathetosis, dystonia) and muscle tone (hypertonia, hypotonia), REE has been reported to be significantly lower in most children with CP when compared to neurologically normal children. Conversely, it has been hypothesized that children with athetosis have similar or even increased ERs compared to recommendations for neurologically normal children, because of increased involuntary movements at rest. When considering functional ability, nonambulatory children have significantly lower REE levels than do children with greater function. An accurate estimate of ERs is important for nutritional intervention, but it is often difficult to obtain in NI children. Currently, equations specific for CP children are available. The first equation was developed by Krick *et al.* in 1992 [24], more recently Rieken *et al.* [25] developed two equations for use in nonambulant, school-age children with severe CP. One requires prediction of basal metabolic rate with the commonly used Schofield equation [26], and the other equations use a multiple of total body water measurements. From these baseline calculations, each equation then estimates the total energy expenditure with corrections for physical activity levels, gross motor function level (GMFCS) and a general correction for the existence of CP. In a study conducted by Walker *et al.* in 32 pre-school children with CP aged 2.9–4.4 years, ERs were calculated using the doubly labeled water method. The study showed that ERs decreased as ambulatory status declined and more limbs were involved. The greatest predictor of ERs was fat-free mass, then ambulatory status [27].

### 2.4. Gastrointestinal Disorders Affecting Nutrient Intake

The most frequent gastrointestinal disorders that impact on feeding dynamics and significantly affect dietary intakes in NI children are dysphagia and gastroesophageal reflux [28,29,30]. In addition, constipation is a frequent complication in children with neurological impairment and when it occurs on a regular basis and/or becomes severe, it can impact on eating habits and nutrient intake, due to abdominal pain, nausea and vomiting [29]. The appropriate control of the digestive system depends on the healthy functioning and integrity of the neural system. Patients with structural abnormalities of the central and peripheral nervous systems are more likely to develop gastrointestinal and eating disorders.

### 2.5. Dysphagia

Dysphagia, occurs as a result of impairment of one of the phases involved in the swallowing process: oral, pharyngeal or esophageal phases. The foregut, from mouth to duodenum, is the part of gastrointestinal tract most severely affected in children with CP, because of its great density of extrinsic innervations, which are damaged by the initial injury to the central nervous system. Dysphagia is common in children who acquire brain damage in early in life [13,31], for example in children with CP, but may also occur in children with brain injury acquired later in life [32,33] (e.g., trauma, stroke, encephalitis, and brain tumor), in children with genetic disorders including Down’s and Rett syndrome [34,35,36] or in children with neurological degeneration such as muscular dystrophy [37].

Oropharyngeal dysphagia (OPD), which includes impairment of both the oral and pharyngeal phases, is one of the major factors involved in etiopathogenesis of poor feeding and reduced dietary intakes in NI children. In fact, OPD is responsible for inefficient feeding process due to excessive food spillage, which is not available for energy and nutrient needs. It causes children to eat more slowly than other members of the household, taking up to 2–12 times longer to swallow pureed food and up to 16 times longer to chew and swallow solids compared to healthy children. As a result, regular family or school mealtimes may be insufficient for these children, leading to underfeeding and total dependency on a caretaker.

It is believed that OPD is highly prevalent in individuals with CP; however, the true prevalence is difficult to define due to the lack of well-conducted population based studies. Estimates vary significantly according to the literature from 19% in a large register sample (*n* = 1357 children with CP, aged 5–11 years) [38] to 99% in a smaller sample of children with moderate-to-severe gross motor impairment (*n* = 166, aged 2–19 years) [39]. Variability in prevalence could be due to methodology limitations (parent reports, use of non-validated methods, and case-definition of OPD), and inclusion of individuals with a broad range of gross motor impairment and different ages.

One of the first studies assessing oral motor skills in children with CP was conducted by Reilly *et al.* [13]. The Authors evaluated oral motor skills by means of a video recording in 49 children with CP aged 12–72 months in community. The study found that 90% of subjects had clinically significant OPD; one in three of these children had such severe OPD that their oral intake was restricted to either liquids or pureed foods. More than half of subjects (60%) were totally dependent on their mother for all aspects of feeding, 57% presented with choking episodes which required medical attention at least once as infants and 71% presented with frequent coughing and choking. The severity of OPD was directly correlated to severity of functional motor impairment, with moderate-to-severe OPD occurring mainly in children with tetraplegia. Early feeding histories of these children revealed that in a significant proportion of children, feeding problems occurred within the first 12 months of life, preceding the diagnosis of CP in many cases, confirming the hypothesis that feeding behavior is a sensitive indicator of central nervous system integrity in neonates [40]. During the subsequent years, several studies have focused on OPD and specifically on its relation with gross motor function level in children with CP [41,42,43,44,45,46,47]. Overall, data have shown consistent results supporting a direct correlation between severity of OPD and the level of gross motor impairment, evaluated by Gross Motor Function Classification Scale (GMFCS).

Recently, Benfer *et al.* aimed at defining the prevalence and clinical signs of oral and pharyngeal phase impairments in CP children with OPD in two separate studies [48,49]. In 130 children diagnosed with CP at 18–36 month, with GMFCS I-V, the oral phase was studied by means of a video and with the Dysphagia Disorders Survey Schedule for Oral Motor Impairment. Overall, 93.8% of children were identified with oral phase impairments during eating or drinking, or in controlling saliva. Difficulty biting (70%), cleaning behaviors (70%) and chewing (65%) were the most frequent signs on solid foods and difficulty sipping from a cup (60%) for liquids [48]. With regards to pharyngeal phase impairment, in the study conducted by Benfer *et al.* [49] more than half of subjects (68%) had clinical signs suggestive of pharyngeal phase impairments. The most common signs on direct-assessment were coughing (44.7%), multiple swallows (25.2%), gurgly voice (20.3%), wet breathing (18.7%) and gagging (11.4%). Many of the signs were more commonly observed on fluids, although multiple swallows and gag were more common on solid foods [49]. Identification of pharyngeal phase impairment may be difficult in children with CP due to the lack of coughing reflex reported in 82%–94% of aspiration cases and making children with CP at risk of “silent aspiration” [50].

### 2.6. Gastroesophageal Reflux

Gastroesophageal reflux (GER) is highly prevalent in NI children, occurring in up to 75% of cases [51,52]. Many factors may concur to the etiopathogenesis of GER, including esophageal dysmotility, hiatus hernia, prolonged supine position, increased intrabdominal pressure secondary to spasticity, scoliosis or seizures and also antroduodenal dysmotility. The typical clinical signs of GER are frequent regurgitation and vomiting, which can significantly contribute to nutrient and energy losses. Recurrent episodes of GER, with frequent exposure of the esophageal mucosa to acidic refluxate can lead to complications such as peptic esophagitis, ulceration and stricture formation. Neurologically impaired children are often unable to communicate symptoms such as burning epigastric pain and dysphagia and may manifest food avoidance or aversion, leading to significant reduction in energy and nutrient intakes.

### 2.7. Constipation

Chronic constipation (CC) is a common problem in children with neurodisabilities. Estimates of the prevalence of constipation vary from 26% to 74% in children with severe disabilities depending on the definition of constipation, the diagnostic method, and participant selection [29,30,53]. Contributory factors include abnormal bowel mobility, prolonged immobility, skeletal abnormalities, generalized hypotonia and reduced fluid and fiber intake. If chronic constipation is not adequately treated, it can lead to several gastrointestinal and nutritional complications including chronic nausea, recurrent vomiting, abdominal pain, early satiety with food refusal and poor dietary intake.

## 3. Non-Nutritional Factors

Among the non-nutritional factors impacting on dietary intake and nutritional status in NI children, cognitive impairment and prolonged use of antiepileptic medication are involved.

### 3.1. Intellectual Disability

Mental retardation as well as hearing, language, visual, and behavioral disorders are often associated to neurological impairment. Cognitive impairment may be responsible of inability to communicate hunger or satiety, inability to request food and drink and to communicate symptoms. There is scientific evidence that the prevalence of malnutrition increases with lower intelligence quotients (IQs). In a study conducted by Sánchez-Lastres *et al.* assessing nutritional status in 128 mentally retarded children in north-west Spain, the results showed that children with a state of malnutrition had significantly lower mean IQs than those in the normal nutrition range, with a severity of malnutrition which increased with increasing IQs deficit [54]. These findings are in accordance with other previously conducted studies [55,56].

### 3.2. Antiepileptic Therapy: Gastrointestinal Disturbances and Bone Disease

Children with neurological impairment are often affected by epilepsy and usually require a long-term treatment with antiepileptic drugs (AEDs). Valproic acid (VPA), the most frequently prescribed antiepileptic drug in pediatric age, is usually well tolerated but it is not totally free from adverse effects.

- Gastrointestinal disturbances: Among the dose-dependent adverse effects of VPA, gastrointestinal disturbances have been described [57,58]. The main symptoms are feeding difficulties including anorexia and food refusal. Additionally, nausea, vomiting and dyspepsia secondary to gastric intolerance have been described and a single case of VPA-induced gastritis has been reported. More rarely, diarrhea, weight loss, abdominal cramps and constipation may also occur. The above-described gastrointestinal side effects may contribute to poor nutritional status in NI children.
- Osteopenia: Children with neurological impairment are at increased risk for low bone mineral density (BMD) as a result of multifactorial etiology including poor dietary intake of calcium and vitamin D, poor sun-light exposure, muscular weakness, limited mobility, poor weight bearing and use of antiepileptic medications [59]. A growing body of evidence indicates that patients on long-term AEDs are at increased risk for metabolic bone disease including changes in bone turnover, osteoporosis, alterations in bone quality, and most importantly, fractures [60,61]. This issue is particularly important in pediatric age, as this is a critical periods for skeletal mineralization; peak bone mineral density is achieved by the end of adolescence and determines the risk for pathological fractures and osteoporosis later in life. In addition to antiepileptic treatment, patients with epilepsy are at increased risk for fractures secondary to seizure-related falls. As a matter of fact, fractures are reported to be two to six times more common in patients with epilepsy than in the general population [62]. Some AEDs (for example phenytoin, phenobarbital and carbamazepine) induce the hepatic enzyme cytochrome P-450 (CYP-450), increasing the catabolism of vitamin D and inducing a state of hypovitaminosis D with subsequent hyperparathyroidism, increased bone turnover and reduced bone density [48]. In addition, the use of non-enzyme inducing AEDs and polytherapy have been associated with vitamin D deficiency and osteopenia [63,64]. For example, long-term use of VPA is associated with bone metabolism abnormalities, which include reduced BMD and changes in bone turnover, with a dose-response relation. The effect of VPA cannot be explained by vitamin D metabolism, since VPA is not an inducer of CYP-450 system; it is instead thought to act by stimulating osteoclast activity and causing an imbalance between bone formation and reabsorption, hence contributing to bone loss [65]. The effects of the newer AEDs such as gabapentin, lamotrigine, levetiracetam, oxcarbazepine, topiramate and zonisamide on bone and calcium metabolism need to be better defined due to the limited number of studies conducted in this field.

## 4. Nutritional Assessment and Intervention

**Figure 1:**
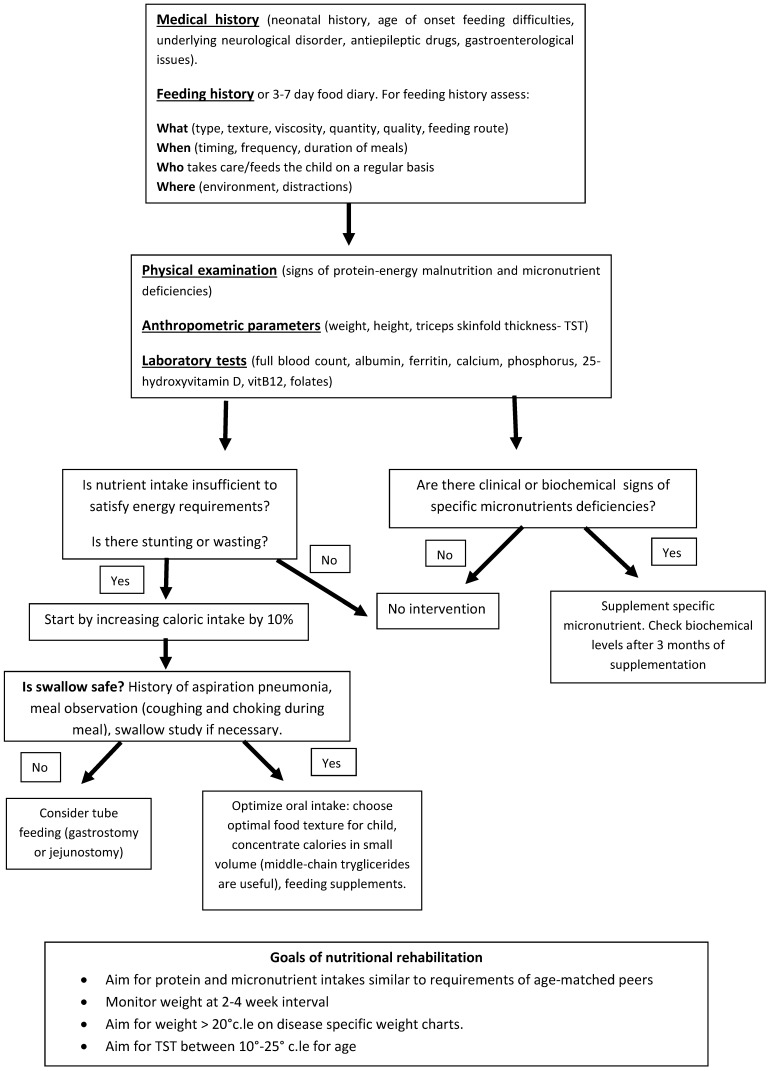
Proposed approach for nutritional assessment and intervention in NI children.

Nutritional support is an essential part of the care of NI children, which have extremely complex and challenging needs. Adequate nutritional support may restore linear growth, normalize weight, decrease irritability and spasticity, improve wound healing and peripheral circulation, reduce the frequency of hospitalization, increase societal participation, hence improve overall health and quality of life. For a successful evaluation and nutritional management of NI children, a multidisciplinary approach is fundamental with the interaction of pediatric nutritionists, gastroenterologists, dietitians and neurologists. A proposed approach for nutritional assessment is illustrated in Figure 1.

Prior to nutritional assessment it is fundamental to collect an accurate medical history including the underlying neurological disease, the level of motor and cognitive impairment, the type and duration of antiepileptic treatment, the number of hospitalizations, the presence and severity of gastrointestinal disorders (OPD, GER and constipation). Nutritional assessment should include anthropometric evaluation and feeding history.

### Nutritional Assessment and Intervention

- Physical examination: Physical examination should search for signs of protein energy malnutrition and for signs of specific micronutrient deficiencies. Skin (color, level of hydration, rashes, decubitus ulcers) and distribution of subcutaneous fat should be accurately observed. Pallor can be suggestive for iron deficiency anemia, decubitus ulcers and poor subcutaneous fat can be suggestive for protein energy malnutrition. Tone and trophism of muscle masses, activity level and athetoid movements should be examined with attention and are important because they influence energy needs. Trophism of nails and hair are also important because they can suggest iron, zinc and copper deficiencies if severely dystrophic. The mouth should also be carefully inspected and specific attention given to the presence of ulcers and gingival bleeding, suggestive for micronutrient deficiencies (vitamin A and C) and to caries which are often present in children with CP due to difficulties in oral hygiene and to gastroesophageal reflux.
- Anthropometrics and body composition. Measuring nutritional status can be challenging in NI children. Weight and height are often difficult to measure due to inability to stand, scoliosis or joint contractures of patients. Segmental measures are a reliable method for obtaining an estimated height for patients who cannot stand. Equations are available to calculate height from ulnar length, knee height and tibial length [66,67,68,69]. Wheelchair scales or weighing a child along with a parent and subtracting parental weights provide a reliable weight if a child cannot stand on a scale. Descriptive growth curves specific for children with gross motor impairment are available and are a reliable tool to assess whether a child is growing adequately, most important is to follow a child over time and make sure he/she is growing along his/her own growth curve [70]. Body mass index (BMI) or weight-for-height are frequently used to estimate nutritional status, yet they are poor predictors of body fat percentage. Additionally, BMI does not allow differentiation between lean and fat mass and has been shown to have only a low to moderate correlation with body fat in individuals with moderate to severe CP [71,72]. Knowledge on body composition is useful as it can help determine the requirement for nutritional intervention; some experts recommend that body composition be measured routinely in nutritional assessment of children with CP [73]. There is ongoing research to find safe, non-invasive, cost-effective, and valid methods to assess the body composition of children with CP. Dual-energy X-ray absorptiometry (DXA) is not always feasible in clinical practice due to cost constraints and availability. Simpler and less expensive methods to assess body composition include skinfold measurements and bioelectrical impedance assessment (BIA). Skinfold thicknesses, in particular triceps skinfold thickness, have long been considered important and valid measurements of subcutaneous fat. However, given a tendency of children with CP to store fat centrally, reduced peripheral skinfold thicknesses may not necessarily mean low fat stores and in addition, no skinfold reference curves specific for CP children are available [74]. Cerebral palsy specific equations have been developed by Gurka *et al.*, these permit to estimate percentage of body fat from two skinfold measurements; their validity is currently being evaluated in ongoing studies [75]. Recently, Oeffinger *et al.* have shown that that BIA and two skinfold measurements (using CP specific equations) are accurate and non-invasive methods to estimate body fat percentage in children with CP [76].
- Feeding history and nutrient intake: The most reliable method to assess dietary intake is by means of a seven-day feeding history collected by an experienced dietitian but this may not be always feasible and available in clinical practice. The majority of scientific literature on dietary intakes in CP children has used a three-day food diary. Recently, a modified three-day weighed food record for measuring energy intake has been validated for preschool-aged children with CP by Walker *et al.* [77]. Nevertheless, it must be noted that dietary histories and food records even though easy to perform, have some limitations for example they might not truly reflect the real dietary intakes of the subject. This is for two reasons, in first instance food diaries might not report the correct frequency and quantity of food consumed by the patient. Secondly, the food composition tables, which are used to calculate the specific macro- and micronutrients, are often inaccurate, in particular with regards to vitamins and minerals. When evaluating NI children for feeding problems, parents/caregivers should pay attention to the time needed to feed the child. If mealtimes last more than 30 min on a regular basis and/or mealtimes tend to be stressful for the child or parents, a referral for a more comprehensive feeding evaluation by a specialized feeding therapist might be useful. If during mealtimes respiratory symptoms such as coughing, choking, gagging, wet/gurgly respiration or rattly chest should occur, confirmation of swallowing impairment is useful with videofluoroscopic swallow study (VFSS). Adequate positioning and physical support, for example head support to avoid hyperextension or flexion, is important to ensure safety of the swallowing process. The feeding history should also take into account the modality of feeding (orally *vs*. tube feeding), the textures of food and thickness of fluids in the diet. Textures of food and thickness of fluids may need to be modified, to ensure airway safety, maximize eating efficiency and reduce fatigue during mealtimes. In case of tube feeding, the type of formula, the route of administration (gastric *vs*. jejunal), the quantity and tolerability should be assessed.
- Laboratory tests: Blood tests are less reliable indicators of nutritional status, there is no single blood biomarker that can identify a status of calorie-protein malnutrition with good sensitivity and specificity. For example serum albumin concentrations are influenced by numerous non-nutritional factors such as inflammatory status (negative acute phase protein), hydration status, fluid distribution and hepatic function. It also has a long half-life (21 days) and hence its serum concentrations decrease slowly in case of malnutrition. Laboratory tests may be useful to identify specific micronutrient deficiencies for example a full blood count and ferritin levels can show iron deficiency anemia. Abnormal serum calcium, phosphorus, alkaline phosphatase and 25-hydroxyvitamin D levels may reflect poor bone mineral status.
- Estimating nutritional requirements: Estimating the nutritional needs of NI children is challenging. Many children with CP have decreased energy requirements (ER) in comparison with neurologically normal children, and these tend to decrease with increasing severity of motor impairment. Energy needs of children with severe CP who are nonambulant have been reported to be between 60% and 70% of neurologically normal children [27,78,79,80]. Participation in physical and social activities may increase the energy requirements of children with CP and need to be taken into account when estimating ER. Equations to calculate ERs of NI children are available and have already been mentioned above [24,25,26]. Even though none of them is perfect and overestimation of ERs is frequent, the Krick method is one of the most reliable as it takes into account mobility, muscle tone, activity level, altered metabolism and growth [24].
- Nutritional intervention: Nutritional intervention should always be tailored on the singular case according to ERs, degree of OPD, safety of swallowing and entity of malnutrition. According to North American Society for Pediatric Gastroenterology, Hepatology, and Nutrition, the indications to start a nutritional intervention are as follows: evidence of oral motor feeding difficulties, undernutrition (weight-for-height <80% expected, BMI <5th percentile), growth failure (height-for-age <90% of expected), overweight (BMI >95th percentile), and individual nutrient deficiencies [81,82]. If oral intake is insufficient to promote weight gain and linear growth, if the amount of time spent to feed the child is excessive because of chewing and swallowing dysfunction, or if aspiration is a risk, enteral tube feeding should be considered. A gastrostomy tube is recommended for long-term enteral nutrition because it is more comfortable for the child and is less easily dislodged than a nasogastric tube. Gastrostomy feedings may promote weight gain, improve the child’s health, and reduce the time spent feeding the child [81,82]. With regards to specific nutrient requirements, unfortunately there are no evidence-based guidelines for nutrient allowances specific for NI children and there is no evidence to support increased protein, vitamin and mineral requirements in this population. The daily recommended intakes (DRI) for typically developing children can be used unless the child is severely undernourished [83]. In the latter case, Kuperminc *et al.* suggest that an intake of 2 g/kg per day of protein and an additional 15%–20% increase of calorie intake may be sufficient to guarantee “catch up growth” [84]. In addition, for micronutrients, standard recommendations of vitamins, minerals and trace elements can be followed with exception of vitamin D, given the increased risk of deficiency due to antiepileptic treatment and insufficient sunlight exposure. Even if there are no consistent clinical data to suggest a dose or formulation for vitamin D supplementation in this population, expert opinion of bone health specialists suggest that a higher daily requirement around 800–1000 UI of vitamin D is safe and should be considered in children with CP [85,86].

## 5. Conclusions

Malnutrition is a frequent complication in NI children impacting on overall health and quality of life. Severity of feeding issues generally increases with reduction of general motor function and cognitive ability. Nutritional assessment and support should be an integral part of the care of NI children aiming at early identification of children at risk of nutrition-related comorbidities. To ensure success of interventions, close monitoring of nutritional status should be performed by a multidisciplinary team.
